# The complete chloroplast genome of *Notholirion bulbuliferum*, lights into phylogenetic and taxonomic analyses

**DOI:** 10.1080/23802359.2019.1696243

**Published:** 2019-12-09

**Authors:** Jie Li, Yixin Liu, Qingjiang Wang, Xuqing Chen, Fengping Yang, Xiuhai Zhang, Yunpeng Du

**Affiliations:** a Beijing Key Laboratory of Agricultural Genetic Resources and Biotechnology, Beijing Functional Flower Engineering Technology Research Center, Beijing Agro-Biotechnology Research Center, Beijing Academy of Agriculture and Forestry Sciences, Beijing, China;; b College of Landscape and Ecological Engineering, Hebei University of Engineering, Handan, China

**Keywords:** *Notholirion bulbuliferum*, complete chloroplast genome, next generation sequencing, phylogenetic analysis

## Abstract

*Notholirion bulbuliferum* a precious endangered species in China, it has a long history of growth and high ornamental and edible value. In this study, we reported a complete chloroplast genome of *N. bulbuliferum*, in which the whole genome is 153,019 bp in length, and includes one large single copy region of 70,585 bp, one small single copy region of 17,527 bp, and a pair of inverted repeat region of 26,530 bp. It contains 130 genes, comprising 84 protein-coding genes, 36 transfer RNA, and eight rRNA genes. The overall AT content of the chloroplast genome is 62.9%. In the maximum-likelihood and phylogenetic analysis with the reported chloroplast genomes of *Notholirion*, it showed that *N. bulbuliferum* and *Notholirion macrophyllum* get a high support rate (ML (BS) =100%) and become sister groups. It indicates that the study on the complete chloroplast genome of *N. bulbuliferum* is more closely related to *Cardiocrinum* than related to *Lilium.*

*Notholirion bulbuliferum* is one of the species of genus *Notholirion* (Liliaceae). It is mainly distributed in Sichuan, Yunnan, Tibet, Shanxi, and Gansu Provinces and grows in the mountain clump (Gao et al. 2010; Yun et al. 2013). The external morphology of *N. bulbuliferum* is characterized by narrow bulbs, black membrane on the outside. The stigma is listed as three petals, and it has basal leaves, flowers standing upright or spreading, and the small bulb is used as a medicine to stop the pain and coughing (Baker 1870; Stearn 1950; Liang 1995). *Notholirion bulbuliferum, Notholirion campanulatum,* and *Notholirion macrophyllum* distributed in China. Unfortunately, largely due to anthropogenic overharvesting and loss of natural habitat, the wild resources of *N. bulbuliferum* have been dramatically decreased. Obtaining genomic data can better help us protect and develop its valuable germplasm, molecular identification, and phylogenetic studies of other species in the genus *Notholirion* (Tiwari et al. 2010). Simultaneously, this is useful for the conservation and exploitation of this valuable germplasm and the molecular identification and phylogenetic study with other species in *Lilium* and *Fritillaria*. The sequence was deposited in GenBank with the accession number MN509268. We assembled and annotated the complete chloroplast genome of *N. bulbuliferum* from next generation sequencing data.

A wild individual sample of *N. bulbuliferum* was collected from Daocheng city (Geospatial coordinates: N: 27°54′22″E：99°57′7″, Altitude: 3636 m) in Sichuan Province, China, and DNA was stored at the herbarium of Institute of Botany, CAS (Herbarium number: BOP127355). Total chloroplast genomic DNA was extracted from the fresh leaves of *N. bulbuliferum* and the total genomic DNA was extracted by the DNAsecure Plant Kit (Aidlab). An Illumina paired-end library was prepared and used for Next Generation Sequencing on the HiSeq4000 Sequencing System at Novogene (http://www.novogene.com/index.php), Beijing, China. A genomic DNA library was constructed using VAHTSTM Turbo DNA Library Prep Kit for IlluminaVR (Vazyme, Nanjing City, China). Then, top-quality reads were produced to map the whole genome using the program sequencher 5.0 (Gene Codes Corporation, USA), with that of reported chloroplast genome of Liliaceae (Tiwari et al. 2010; Du et al. 2016; Hwang et al. 2016; Lu et al. 2016) as the reference. The gene annotation was performed with the online program Dual Organellar GenoMe Annotator (http://dogma.ccbb.utexas.edu/) (Wyman et al. 2004). A physical map of the genome was drawn using OGDraw v1.2 (Lohse et al. 2013).

The whole genome of *N. bulbuliferum* is 153,019 bp in length; within, the genome includes a large single copy (LSC) region of 70,585 bp, a small single copy (SSC) region of 17,527 bp, and a pair of inverted repeat (IRA and IRB) regions of 26,530 bp. This genome composition is asymmetric (31.1% A, 18.9% C, 18.2% G, and 31.8% T). The A + T content is 62.9%, C + G content of 37.1%. The complete chloroplast genome contains 130 genes, comprising 36 transfer RNA genes and eight ribosomal RNA genes and 84 protein-coding genes. In addition, 18 genes (*trnK-UUU*, *rps16*, *atpF*, *rpoC1*, *ycf3*, *trnL-UAA*, *trnV-UAC*, *clpP*, *petB*, *petD*, *rpll6*, *rp12*, *ndhB*, *rps12*, *trnI-GAU*, *ndhA*, *trnA-UGC*, *ndhB*) contains single intron, whereas another two genes *(clpP* and *ycf3*) all have two introns, four genes (*trnA-UGC*, *trnI-GAU*, *ndhB*, *rp12*, *rps12*) are located within the IR regions.

In order to make sure that phylogenetic relationship between *N. bulbuliferum* and its related species, a maximum-likelihood (ML) phylogenetic tree was constructed with CIPRES (http://www.phylo.org/) (Miller et al. 2010). The phylogenetic tree divided the 16 species, 10 species belonging to the *Lilium,* one species belonging to the *Notholirion*, two species belonging to *Cardiocrinum*, and two belonging to *Fritillaria* (Figure 1). In the ML tree, *Fritillaria* as outer group, the results show that *N. bulbuliferum* together with *N. macrophyllum*, which are sister relationships, and each nodes were supported by bootstrap values 100%. Moreover, based on the sequence of the complete chloroplast genome, it indicates that the study on the complete chloroplast genome of *N. bulbuliferum*, which is more closely related to *Cardiocrinum* than related to *Lilium.* It provides reference data for the identification, classification, and phylogenetic analysis of Liliaceae in the future. Simultaneously, it also provides information for *Notholirion*, *Cardiocrinum*, and *Lilium* chloroplast genome research.

**Figure 1.**
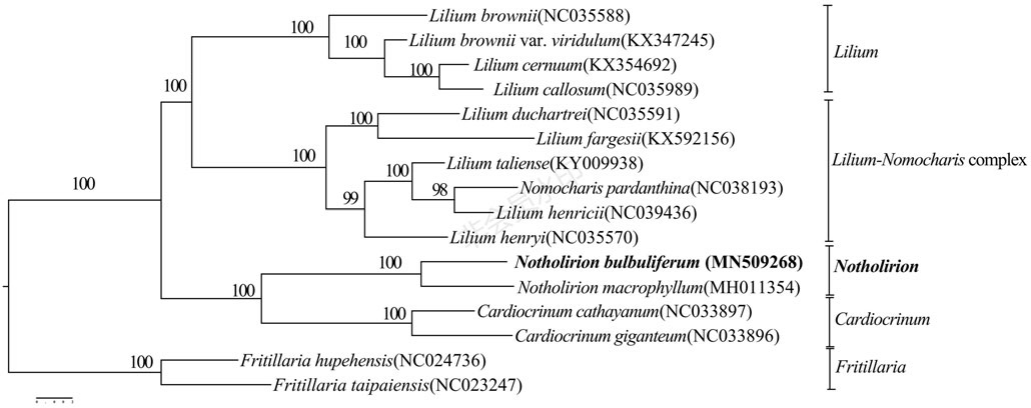
Phylogenetic relationships of *Notholirion bulbuliferum* with the outgroup of *Fritillaria* constructed by whole chloroplast genome with the maximum-likelihood (ML) analyses. The bootstrap values were based on 1000 replicates.
